# Radiological Reporting of Urgencies Related to Medical Devices: Commentary on a Possible Systematic Approach

**DOI:** 10.3390/tomography7030024

**Published:** 2021-06-29

**Authors:** Andrea Contegiacomo, Marco Conti, Massimo Muciaccia, Pietro Trombatore, Michele Dezio, Emilio Lozupone, Agostino Meduri, Riccardo Marano, Luigi Natale, Riccardo Manfredi

**Affiliations:** 1 Fondazione Policlinico Universitario A. Gemelli IRCCS, 00168 Rome, Italy; andrea.contegiacomo@gmail.com (A.C.); marco.conti@guest.policlinicogemelli.it (M.C.); Agostino.Meduri@unicatt.it (A.M.); riccardo.marano@unicatt.it (R.M.); luiginatale@mac.com (L.N.); riccardo.manfredi@policlinicogemelli.it (R.M.); 2 Department of Diagnostic Imaging, Oncological Radiotherapy and Hematology, Università Cattolica Sacro Cuore, 20123 Milano, Italy; pietro.tr@outlook.it; 3 Ospedale Madonna delle Grazie, 75100 Matera, Italy; dezio.michele@virgilio.it; 4 Ospedale Vito Fazzi, 73100 Lecce, Italy; emilio.lozupone@live.it

**Keywords:** medical device, complication, emergency department, CT, X-ray, radiology checklist

## Abstract

Most medical devices are routinely recognized on radiological images and described as normal findings in the radiological report, but sometimes they can cause patient access to the emergency department. Multiple possible complications have been described and most of them require prompt recognition by radiologists for proper clinical management. This commentary proposes a systematic approach to radiological reporting of the most common emergent complications related to medical devices with the intent to avoid the omission of important findings in the final radiological report.

## 1. Introduction

Medical devices are defined as any instrument, tool, apparatus, appliance, software or material used specifically for diagnostic and/or therapeutic purposes for human beings [1]. In some cases, medical devices can cause dramatic clinical events, requiring a fast diagnosis and intervention. Although advanced identification techniques have been proposed [2], radiologists still play a central role in the diagnostic workup of emergencies associated to medical devices. The radiological task appears to be extremely challenging due to the great variability of possible clinical and imaging scenarios, and to the lack of evidence in the literature on the reporting process.

This commentary proposes a systematic approach for radiological reporting of medical device emergencies, aiming to avoid the omission of important information for clinicians and to ensure the most appropriate management for the patient, even in extremely complex situations.

## 2. Device Identification and Recognition

**Figure 1:**
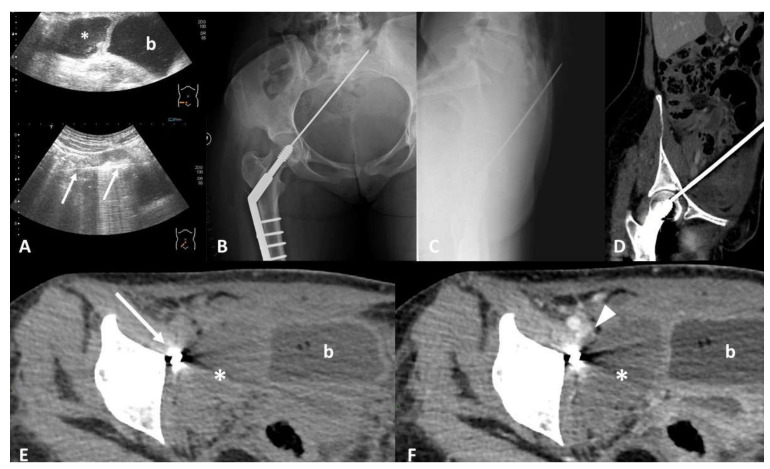
Device identification and recognition. Twenty-nine-year-old female with acute abdominal pain after surgical repair of a traumatic femoral neck fracture. Ultrasound scans (**A**) show a hypoechoic collection (*) in the right iliac fossa, close to the bladder (b), and a linear hyperechoic image with sonographic artifacts (arrows). Frontal (**B**) and lateral (**C**) radiographs show a sharp portion of the osteosynthesis passing through the pelvis, with the tip into the soft tissue of the anterior abdominal wall. Computed Tomography (CT) multiplanar reconstruction images better show the course of the misplaced device from the femoral head to the anterior abdominal wall (**D**). Non-enhanced CT scan (**E**) confirms a large collection (*) in the right iliac fossa, next to the misplaced device (arrow), characterized by hematic density. Contrast-enhanced CT images (**F**) better define the relationship between the collection (*) and the iliac vessels, showing a hypodense filling defect (arrowhead) inside the right external iliac vein (EIV), suggesting the diagnosis of a hematic collection due to iatrogenic injury of the EIV caused by the misplaced device.

The identification and recognition of a medical device is the first step. Patient clinical history should be examined thoroughly to achieve this goal. Detailed information about implant or utilization time and modality, and device brand, shape, material, and expected location should be provided by clinicians and the patient himself. All these factors are useful in choosing the most appropriate imaging modality for device identification and recognition, improving the radiologist’s diagnostic accuracy, and reducing time between diagnosis and treatment (Figure 1).

Chest and abdominal radiographies can help in the identification of radiopaque devices [3] and usually assist the radiologist in selecting further, more invasive, diagnostic investigations. If the radiography fails to identify a radiopaque device, the possibility of a spontaneous expulsion should also be investigated [4,5]. Ultrasound examination is an option to consider before moving to diagnostic modalities that increase ionizing radiation exposure, especially in pediatric and pregnant patients, or in the case of superficial/subcutaneous position of the devices.

## 3. Device Integrity and Migration

**Figure 2:**
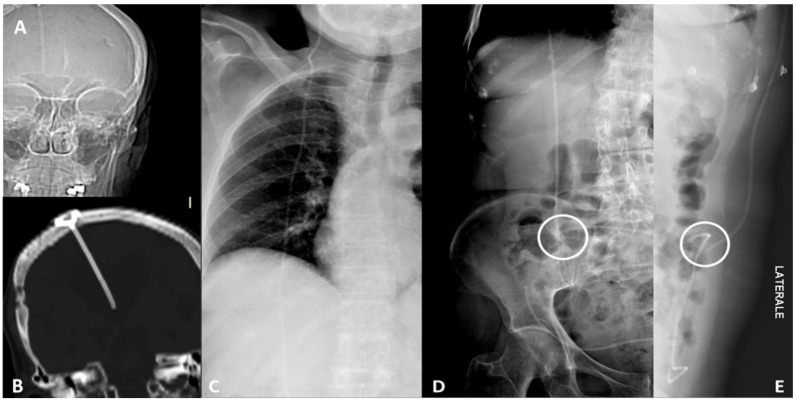
Device integrity. Ventriculoperioteneal shunt malfunction in a patient with history of hydrocephalus. CT scout view (**A**) and coronal CT scan (**B**) of the head show the correct placement of the tip of the shunt inside the right lateral ventricle. Frontal chest radiograph (**C**) shows the integrity of the device along its thoracic course. Frontal (**D**) and lateral (**E**) abdominal radiographs detect a fracture of the device in the right lower quadrant of the abdomen.

**Figure 3:**
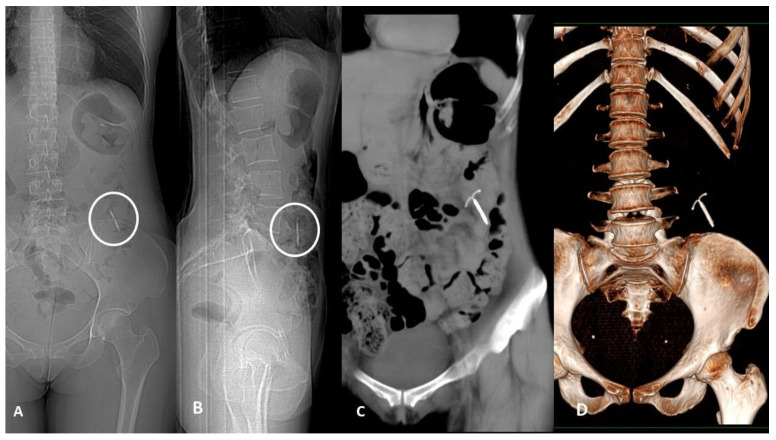
Device migration. Thirty-nine-year-old woman with acute left flank pain and history of copper Intra-uterine device (IUD) insertion 2 years earlier. Frontal (**A**) and lateral (**B**) CT scout of abdomen identify the IUD in the left lower quadrant. Non-enhanced CT scan coronal plane MIP-reformatted (**C**) shows device dislocation medially to descending colon in the pericolic fatty tissue; no displacement-related complications such as pneumoperitoneum or fluid collection were found. CT 3D-reconstruction (**D**).

**Figure 4:**
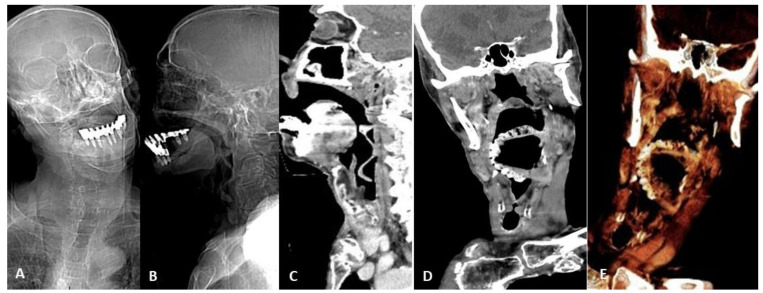
Device migration. Eighty-seven-year-old man affected by Alzheimer disease and asphyxiation crisis. Frontal (**A**) and lateral (**B**) head and neck CT scout views show denture migration with hypopharynx impaction. Contrast-enhanced CT sagittal (**C**) and coronal (**D**) scans better show denture relationship with the surrounding structures and demonstrate anterior epiglottis displacement with subsequent airways restriction (arrow). 3D VR image thin slab (5 mm) view (**E**) better shows device migration.

**Figure 5:**
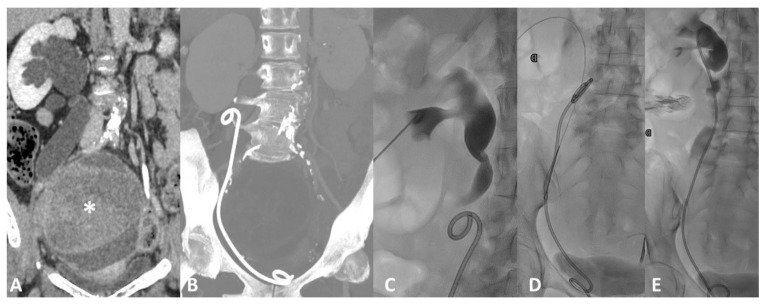
Device migration. Forty-six-year-old female with rapid onset of right flank pain ad diuresis reduction 3 months after ureteral stent placement for marked hydroureteronephrosis (**A**) caused by an ovarian mass (*). Follow-up CT scan (**B**) shows displacement of the ureteral stent, slipped down in the urinary tract. Fluoroscopic image (**C**) shows the consequent dilatation of the renal pelvis and calyces with stasis of contrast medium caused both by the malfunction of the displaced device and by the mechanical obstruction caused by the stent itself inside the ureter. Percutaneous Stent retrieval with a goose neck snare catheter (**D**) and replacement (**E**) was the treatment of choice.

Once the device has been identified, all of its components must be recognized and their integrity (Figure 2) and location (Figure 3) assessed. Any fracture [6] and/or migration [7] of the device or its components should be reported and described in detail; in case of fracture, the number of fragments and their location should also be reported in order to plan either a retrieval attempt or a conservative management, as in the case of gastrointestinal devices that could be self-expelled. A detailed description of its relationship with the surrounding anatomy is essential, especially if vital structures are involved (Figure 4); moreover, it is important to investigate the consequences of a malfunction of the damaged/displaced device. Suggestions should be given on the possible retrieval technique such as endoscopy, interventional, or surgery (Figure 5).

## 4. Complication Reporting

Identification and definition of complications is probably the most important part of the radiological task. This step is essential to direct the patient towards the best therapeutic management in the optimal time frame and can significantly affect the final outcome.

The majority of device-related complications can be grouped into five different categories, which can occur individually or in combination in the same patient.

### 4.1. Vascular Injury

**Figure 6:**
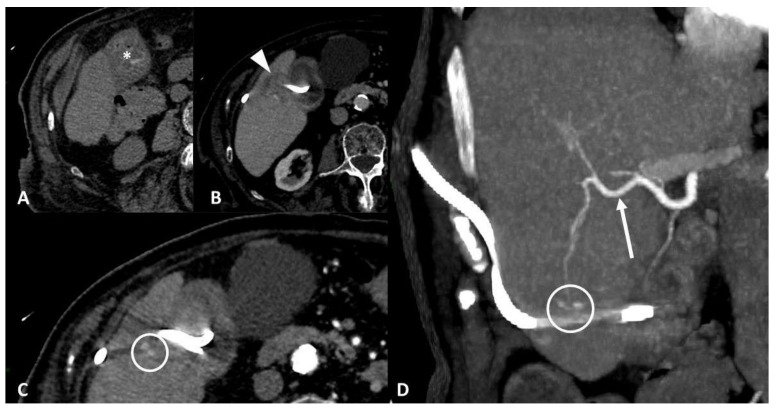
Vascular injury. Seventy-five-year-old patient affected by bleeding after percutaneous cholecystostomy. Axial non-enhanced CT scan (**A**) shows hyperdense blood-like material inside the gallbladder (*). Contrast-enhanced CT scans (**B**,**C**) show liver parenchymal laceration (arrowhead) in segment V, next to gallbladder, with two hyperdense spots inside. Maximum intensity projection coronal image (**D**) shows more clearly two pseudoaneurysms of a distal branch of the right hepatic artery (arrow) along drainage course.

Vascular injuries are the consequence of a pathological, usually mechanical, interaction between the device and one or more vessels. The vascular wall can be involved in different possible scenarios [8]: Bleeding occurs when the vessel wall is completely damaged and the blood blows out without any containment offered by the surrounding structures; in other cases, tissues restraint leads to the formation of a pseudoaneurysm (Figure 6). Less frequently, the creation of a pathological communication between arterial and venous vessels (arteriovenous fistula) is possible. In all mentioned cases, vascular wall injury is complete.

The device sometimes induces an incomplete wall injury with damage of the intimal layer alone and subsequent vessel dissection or intramural hematoma; in these cases, the activation of the blood coagulation cascade could lead to intraluminal thrombosis, with an increased risk of ischemic damage to distal tissues.

In all cases, information about the vascular anatomy (normal vasculature, anatomic variants, vascular chronic obstruction, tortuosity, collateral circulation, etc.) should always be provided, in order to address patients to the best treatment option.

### 4.2. Parenchymal/Tissue Injury

**Figure 7:**
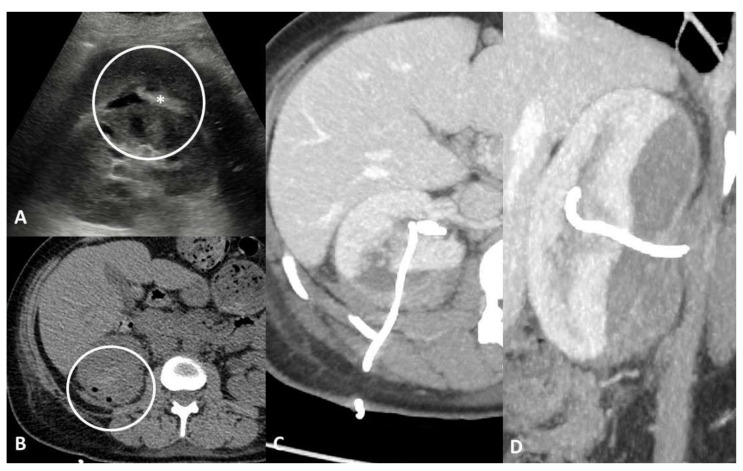
Parenchymal/tissue injury. Forty-one-year-old female with persistent haematuria after percutaneous nephrostomy placement. Axial ultrasound scan (**A**) shows hypoechoic collection of the right kidney with hyperechoic material inside (*). Non-enhanced CT scans (**B**) confirm the presence of a subcapsular hematic collection associated with a heterogeneous density area in the posterior renal parenchyma. Axial (**C**) and coronal (**D**) maximum intensity projection CT images show more clearly the large parenchymal laceration and the direct relationship with the nephrostomy tube, without signs of active bleeding.

All medical devices interact with the surrounding tissues with different modalities according to their shape and, when present, to the strength of impact. Tissue laceration and parenchymal fractures are more frequent when the injury is induced by a device with a sharp morphology, such as catheters or needles [9] (Figure 7), and in the case of strong impacts. When the device has a rounded morphology, penetration is less common; however, the kinetic energy transmitted during impact may lead to the formation of contusive areas or hematomas in the injured tissue.

### 4.3. Obstruction

**Figure 8:**
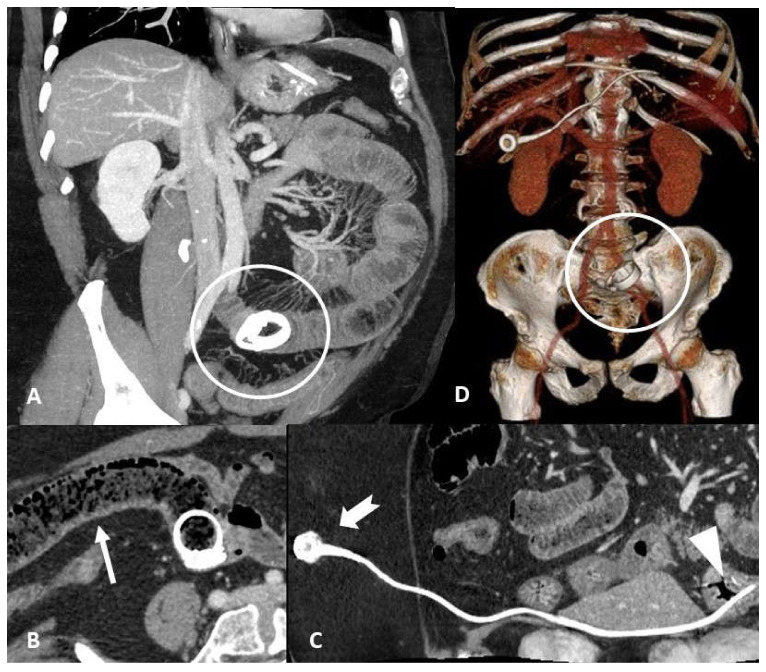
Obstruction. Sixty-four-year-old male with a 3-day history of abdominal pain, constipation, vomiting, and a history of surgery with sleeve gastrectomy and LAGB (laparoscopic adjustable gastric banding) insertion for morbid obesity. Contrast-enhanced CT maximum intensity projection images show (**A**) jejunum dilatation with transition point in mesogastrium caused by gastric band displacement; (**B**) marked dilation of the upstream loop by fecaloid material stasis (arrow) indicates mechanical obstruction. Contrast-enhanced CT multiplanar reconstruction scans (**C**) show port localization in the subcutaneous tissue of the anterior abdominal wall (notched arrow) and the tip of the connecting tube in the gastric cavity (arrowhead). CT 3D-reconstruction (**D**) better shows device disconnection, migration, and integrity of the two components.

Visceral obstruction occurs when a device induces the occlusion of the visceral lumen. The mechanism can be twofold: intraluminal or extraluminal. Intraluminal occlusion occurs when an intraluminal device or its fragment migrates from the operative site to a distal location where the visceral lumen is small enough to halt its progression (Figure 8), or if the content of the viscera is too viscous to get past the device regularly [10]. Extraluminal occlusion is usually the consequence of an extrinsic compression of the viscera by devices implanted either for the injured organ or for an adjacent one. The obstruction mechanism is defined as mechanical if the device compression directly induces the luminal occlusion; if the occlusion is a consequence of luminal narrowing and subsequent stasis of luminal content, the mechanism can be defined as functional.

### 4.4. Perforation

Perforations are caused by a penetrating injury to the wall of hollow organs. The mechanism of injury can be either acute or chronic. Acute perforation usually occurs during a medical procedure [11] or as a consequence of a rapid change in device position. Chronic perforation depends on the pressure induced by the device on the visceral wall, which causes progressive ischemic sufferance, with consequent parietal thinning and, finally, yielding [12].

**Figure 9:**
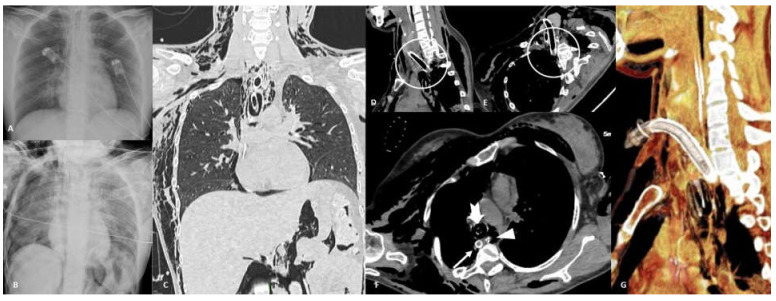
Perforation. Thirty-six-year-old female affected by acute leukemia who underwent tracheostomy placement. Frontal chest radiograph before (**A**) and after (**B**) tracheostomy procedure show the development of right pneumothorax, pneumomediastinum, pneumoperitoneum, and soft tissue emphysema onset. Coronal non-contrast CT scan in lung window (**C**) confirms all these findings. CT unenhanced multiplanar reconstructions (**D**,**E**) show posterior tracheal wall perforation induced by the tip of the tracheostomy tube; CT axial image (**F**) shows displacement of tracheostomy tip in the posterior mediastinum/prevertebral soft tissue (arrow), correct position of nasogastric tube (NSG) in esophageal lumen (arrowhead), and endotracheal tube correctly cuffed (notched arrow). CT 3D-reconstruction (**G**) better shows tracheostomy tube malposition.

Visceral content leakage and fluid collection formation is a common event, and free air can be present when airways or gastrointestinal tracts are involved (Figure 9). The perforation site is usually along the device’s course and should always be investigated in order to direct and facilitate intraoperative identification and repair. Sometimes the device has already been removed or has moved away from its original position; in these cases, knowing the previous position or reconstructing the possible route on the images can be helpful to identify the wall lesion.

### 4.5. Infective/Inflammatory

**Figure 10:**
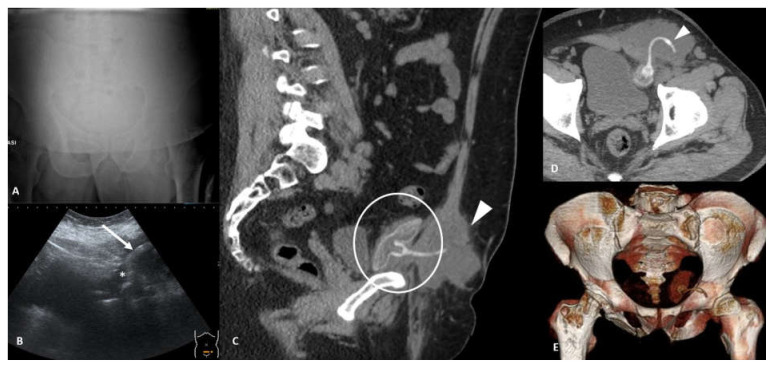
Fluid collection. Fifty-two-year-old male with history of IPP (inflatable penile prosthesis) implantation 6 years ago and recent onset of left groin pain. Frontal orthostatic radiogram (**A**) does not show pneumoperitoneum. Ultrasound pelvic scan (**B**) shows hyperechoic complex image (arrow) in left iliac fossa within a hypoechoic collection (*). Sagittal contrast-enhanced CT (**C**) shows IPP reservoir placed in the retropubic space of Retzius and rectus abdominis tumefaction (arrowhead). Axial non-contrast CT image (**D**) better shows left rectus abdominis tumefaction caused by reservoir saline loss due to disruption of IPP connecting tube (arrowhead); CT 3D-reconstruction (**E**) shows reservoir integrity and connecting tube disruption.

Infectious complications are relatively frequent and can lead to catastrophic consequences such as septic shock and death, if not rapidly corrected. Fluid collections and abscesses are usually appreciated near the device (Figure 10), but distant formation is also possible if the anatomy is favorable, as in the case of intraperitoneal dropping; in this case, the relationship with the device can be less intuitive but a careful clinical analysis usually manages to answer this specific question.

## 5. Final Considerations

Emergencies related to medical devices are insidious and involve different clinical scenarios that can result in a “perfect storm” for radiological images interpretation, dampening the influence of the radiological report on patient. Standardization of radiological report and terminology are valuable tools that can help in preventing interpretative ambiguity. 

**Figure 11:**
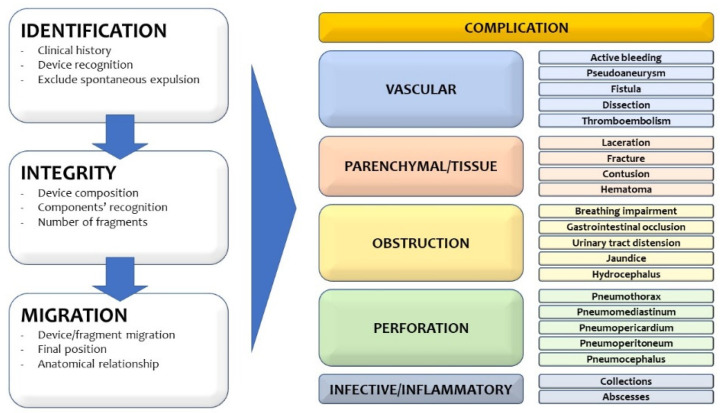
Systematic approach to emergencies related to medical devices.

Furthermore, systematic use of report checklists and of specific search patterns may help to avoid errors or lack of diagnosis. Structured reporting has been advocated as a potential solution to this problematic topic but is beyond the scope of this pictorial essay. It is reasonable that the use of a systematic approach, based on a step-by-step process (Figure 11) as proposed in this essay, could significantly reduce the omission of critical information for the clinicians, especially when multiple findings coexist in the same patient [13,14].

## Data Availability

Images were selected from our institutional database.
